# CNS lymphoma masquerading as stroke: Cases report and literature review

**DOI:** 10.1111/jcmm.17995

**Published:** 2023-11-23

**Authors:** Xiping Liang, Chaoyu Wang, Yao Liu

**Affiliations:** ^1^ Department of Hematology‐Oncology, Chongqing Key Laboratory of Translational Research for Cancer Metastasis and Individualized Treatment Chongqing University Cancer Hospital Chongqing China

**Keywords:** Central nervous system, Hodgkin's lymphoma, stroke

## Abstract

Central nervous system (CNS) involvement in Hodgkins lymphoma (HL) is extremely rare. There are only two reported case series of intracranial involvement of HL. CNS HL can be presented at any point in the course of HL, most mimicking with a prominent neurological symptom. This challenges the diagnosis of CNS involvement and stroke. Here, we report four cases of patients having refractory HL with CNS involvement to garner attention among neurologists for this rare disease presents with stroke symptoms and reviews its disease characteristics, prognosis, and treatment.

## 1 INTRODUCTION

Hodgkin's lymphoma (HL) most commonly presents with progressive, painless enlargement of peripheral lymph nodes, especially around the cervical region or less frequently in the mediastinal or abdominal lymph nodes, and further progresses to other groups of lymph nodes and may spread to any organ system, commonly displaying extra‐nodal involvement including cardiac, pulmonary, hepatic, thymic, splenic and bone infiltration. However, central nervous system (CNS) involvement by HL is extremely rare, accounting to 0.02%–0.5% of HL cases.^1^ In contrast, CNS involvement can occur in 5%–30% of patients with non‐Hodgkin lymphoma.^2^ CNS HL can present at any point in the course of HL, most commonly during relapsing phase and mimics with a prominent neurological symptom.^3^, ^4^

Because of the rarity of CNS involvement, most studies on CNS involvement in HL are case reports. It is rare to note the patterns in its disease characteristics, prognosis, and treatment. Cerebral parenchymal disease and meningeal involvement with parenchymal lesions can also be detected.^3^ Moreover, it is usually the initial presentation of stroke symptoms, which challenges the diagnosis of CNS involvement and stroke.^4^, ^5^ Here, we report four cases of patients having refractory HL with CNS involvement to garner attention among neurologists for this rare disease presenting with stroke symptoms.

## 2 MATERIALS AND METHODS

We retrospectively reviewed the database of patients with HL treated in our hospital from 2011 to 2021. Patients were included if they had brain parenchymal or meningeal HL confirmed with histological or cerebrospinal fluid (CSF) analysis. Clinical data was accessed via the patient's medical records after approval from the appropriate institutional review board.

We studied 689 patients diagnosed with HL. Four adult HL patients with CNS involvement were diagnosed in our center in the last 10 years.

## 3 CASE REPORT

### 3.1 Case 1

A 58‐year‐old female patient was hospitalized for speech impairment and ataxic gait. She was initially diagnosed with ischemic stroke in the district hospital and was given Ischemic stroke therapy for 1 week. However, there was no improvement in her neurological score. The patient had a history of classical HL (mixed cellularity, EBV‐negative) in clinical stage IVB with lung involvement. She was diagnosed 8 months prior to admission and was undergoing treatment with four cycles of AVDB (adriamycin, bleomycin, vinblastine and dacarbazine) chemotherapy and achieved a sustained complete remission. Moreover, 3 months prior to the admission, she had received two cycles of AVD chemotherapy due to severe lung infection.

**FIGURE 1:**
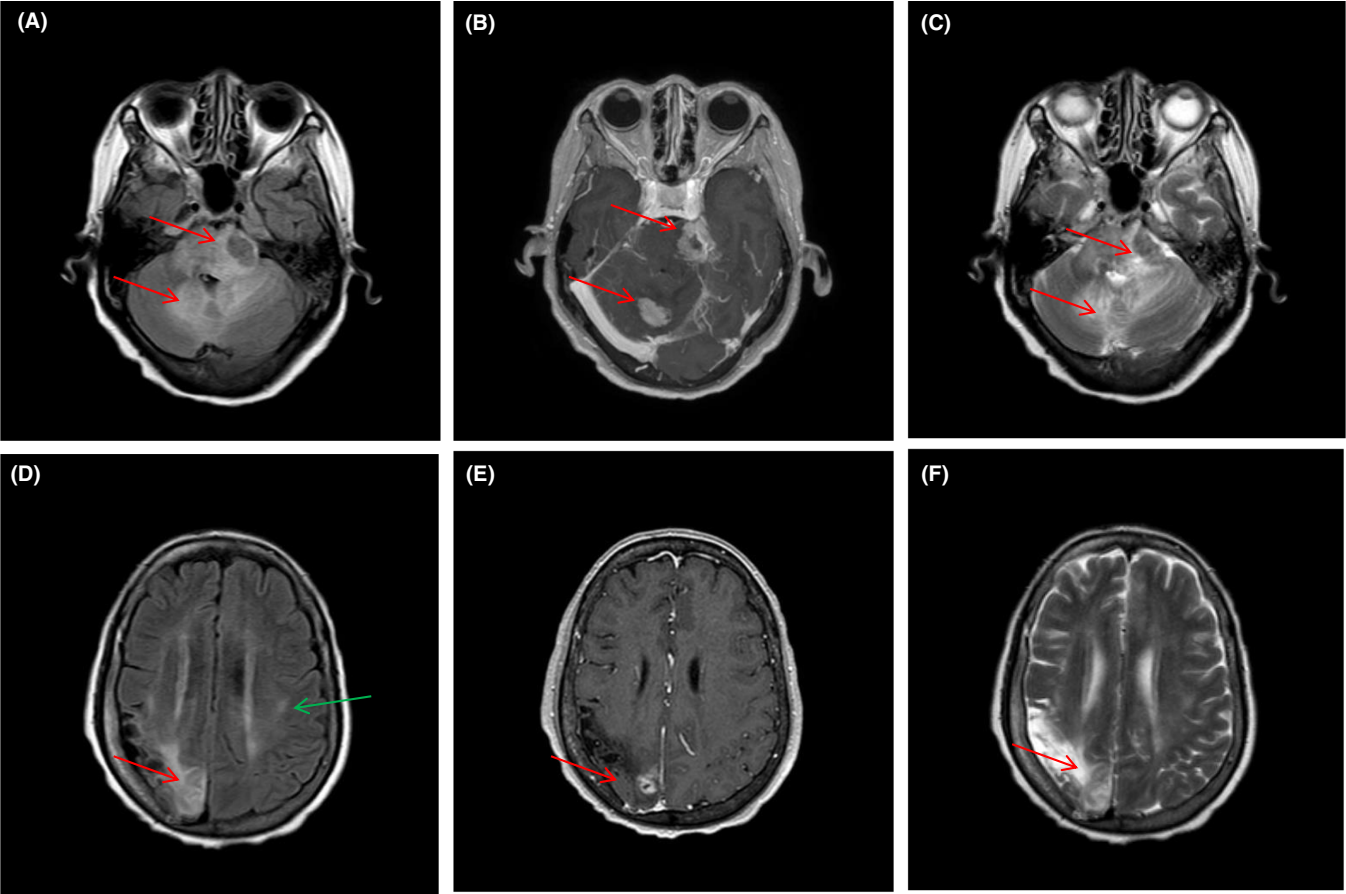
Pre‐treatment magnetic resonance imaging with ill‐defined lesion in brainstem, cerebellum (A, B T1‑weighted axial; C, T2‑weighted axial). T1‑weighted axial (D, E) and T2‑weighted axial (F) images showing enhancing lesion in the cerebral. (Red arrow means NCS involvement, green arrow means ischemia strokes).

Upon examination, vital signs were within the normal range. The patient was alert but could not speak fluently or walk steadily. Blood tests showed mild anaemia of 9.2 g/dL with a normal white blood cell and platelet count. Serum electrolytes, coagulation, liver and renal function tests were normal. Physical examination revealed an impairment in balanced gait experiment, normal cardiac and abdominal exam. To examine a neurovascular cause, we performed an urgent brain MRI (Figure 1), which showed an ill‐defined lesion in the brain stem, cerebellum and bilateral cerebral multiple space‐occupying and prominent vasogenic perilesional edema, and ischemia stroke focus in basal ganglia. Cerebrospinal fluid (CSF) analysis showed mildly elevated proteins with normal protein and glucose. CSF also showed Reed‐Sternberg (RS) cells in this patient (shown in Figure 4A) and negative in other tumour by flow cytometry analysis. However, we did not stain the cells with CD30 or CD15, because there was not enough cerebrospinal fluid to complete the immunohistochemistry. After two cycles of methotrexate + temozolomide chemotherapy, the patient achieved a partial remission. She died of infection 10 months later.

### 3.2 Case 2

**FIGURE 2:**
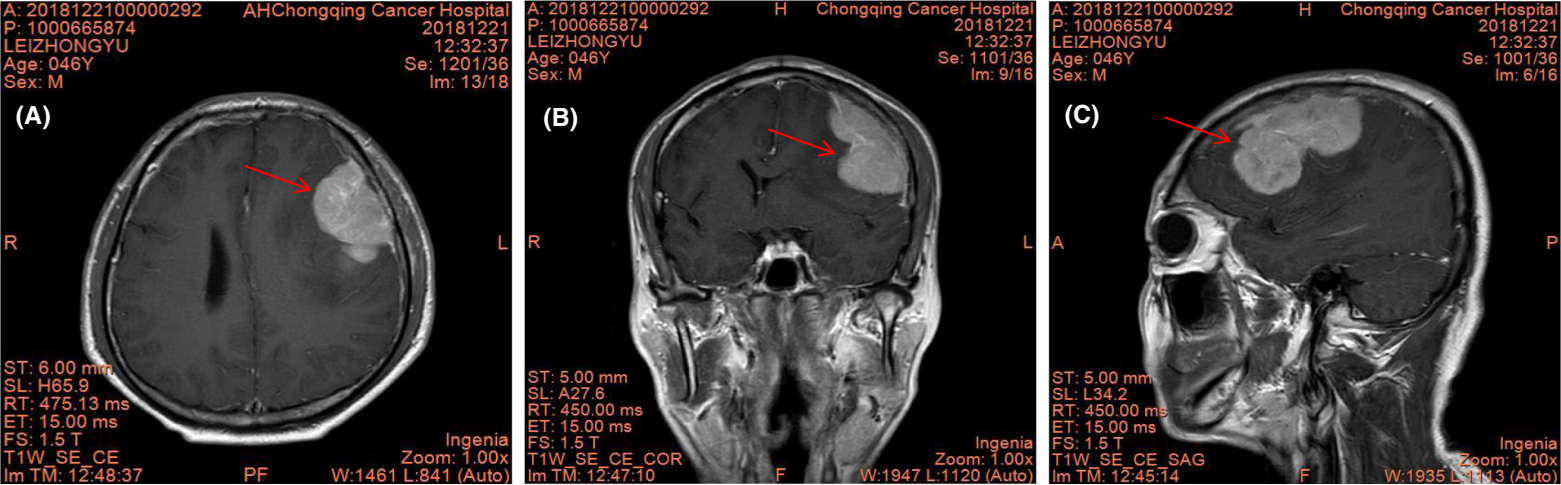
Magnetic resonance imaging show ill‐defined lesion in cranial meningeal and left frontal lobe. (A, Axial T1‐weighted axial; B, T1‑weighted coronal, C, T1‑weighted sagital planes; Red arrow means NCS involvement).

A 47‐year‐old man presented with speech impairment for 1 month, with medical history of middle‐risk stage IIIA classical HL (mixed cellularity, EBV‐negative) 2 years ago. He was treated with six cycles of ABVD chemotherapy in January, 2017, and achieved a complete response. Laboratory examinations revealed normal complete blood cell count and biochemical parameters. Physical examination revealed a normal flat affect, with normal cardiac and abdominal reports. Cranial MRI showed cranial meningeal carcinomatosis and left frontal space‐occupying lesions, and prominent vasogenic perilesional edema considering the possibility of meningeal invasion (Figure 2). Furthermore, lymphomatic brain infiltration was considered in accordance to the medical history.

**FIGURE 3:**
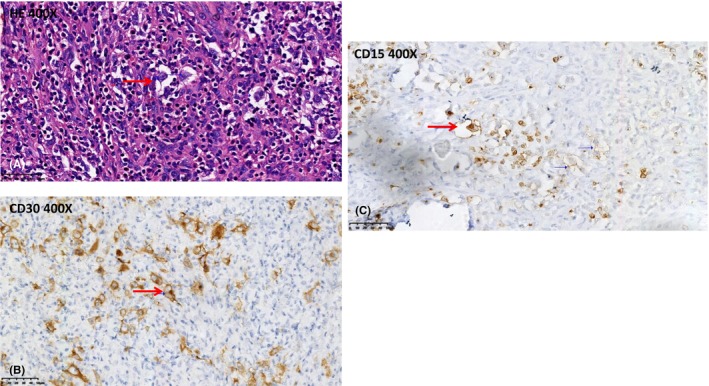
A, Hodgkin (Reed–Sternberg) cells with histiocyte‐rich background (haematoxylin and eosin stain).Haematoxylin & eosin stain at 400× shows a large binucleated cell with conspicuous nucleoli and prominent eosinophilic cytoplasm (arrow), the pathognomonic Hodgkin lymphoma Reed‐Sternberg cell, with positive immunohistochemistry staining (arrows) for (B) CD30 and (C) CD15.

We performed a complete surgical removal of the lesion due to inconclusive neuroimaging findings. Histopathological analysis showed classical HL infiltration in CNS and RS cells, with positive immunohistochemistry staining for CD30, and CD15 (Figure 3), consistent with the diagnosis of HL. After confirmed progression to the CNS, we gave multiple treatments of eight cycles of MTX+GDP (methylamine+gemcitabine+cisplatin+dexamethasone) six cycles of lenalidomide chemotherapy on January, 2019. Up until now, the patient has had no symptoms.

### 3.3 Case 3

A 37‐year‐old male patient was diagnosed with Hodgkin lymphoma in 2015, clinical stage IIIA. He was treated with eight cycles of ABVD chemotherapy, achieving a complete response. Seven years later, he presented with neurologic symptoms of aphasia and weakness in both limbs. He was able to repeat simple phrases with semantic paraphasia and echolalia but could not communicate fluently. The muscle strength in both the lower limbs decreased, and the muscle tension was normal; the rest of the examination was unremarkable.

**FIGURE 4:**
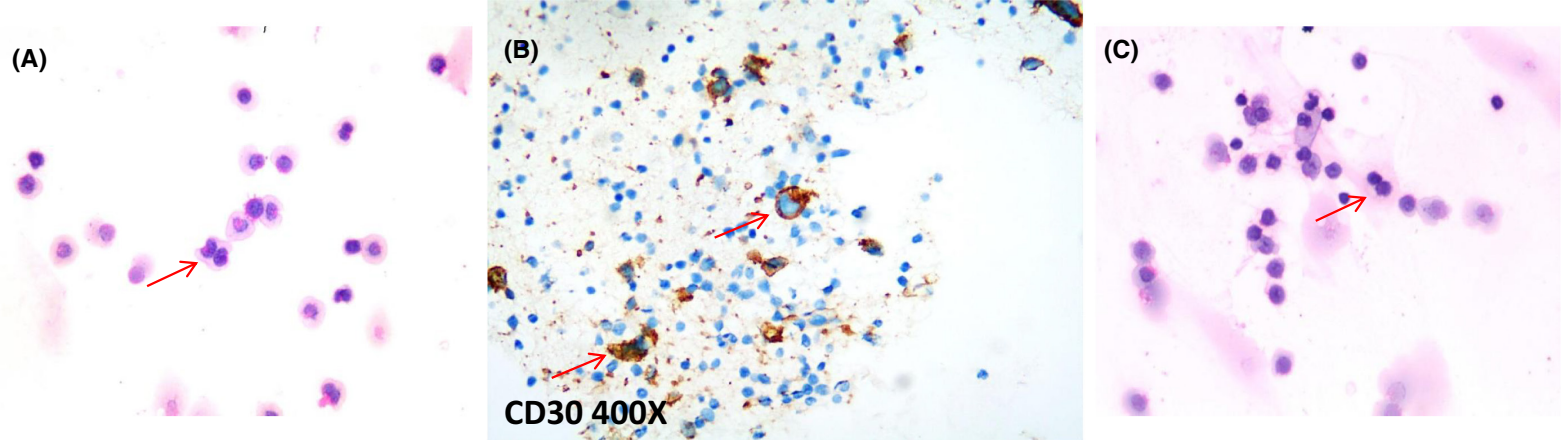
Reed‐Sternberg cells in the Cerebrospinal fluid (CSF), **(**A,C) show Reed‐Sternberg (RS)cell in CSF, (B) show RS cell with positive immunohistochemistry staining for CD30 in CSF.

**FIGURE 5:**
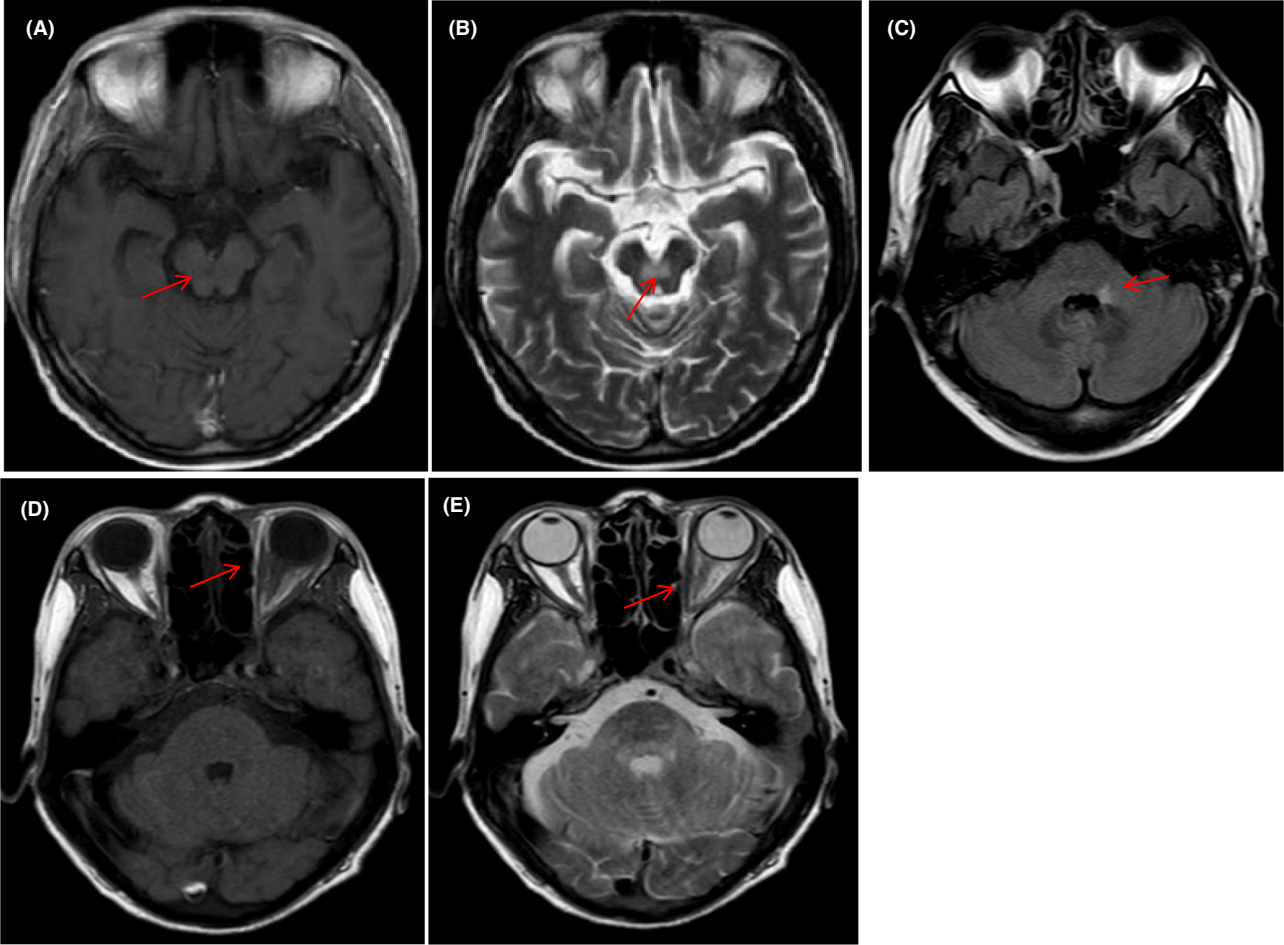
Initial brain MRI shown intracranial extra‐axial lesions on brain stem (A, B) and left pons (C), both hyposignal on T1 WI, T2 WI. (C and D) show ill‐defined lesion in left orbit on T1 WI, T2 WI, respectively.

Serum electrolytes, coagulation, liver and renal function tests were normal. To approach a neurovascular cause, we performed an urgent brain MRI, which showed an ill‐defined, contrast‐enhancing lesion on the left pons, brain stem and bilateral ventricles infiltration and perilesional edema (Figure 5A–C). The CNS involvement by HL was confirmed by CSF cytology. CSF cytology analysis was performed, and large CD30‐positive cells were found (Figure 4B).

Thereafter, we started one cycle of Bv+bendamustine+MTX chemotherapy but no any response was observed. The patient then received two cycles of Teniposide+MTX+PD1 with partial response and improvement in neurologic parameters.

### 3.4 Case 4

A 42‐year‐old man was diagnosed with Hodgkin lymphoma nodular sclerosing subtype in 2018, clinical stage IIIA and lymphocytic predominance subtype. He was treated with ABVD chemotherapy, achieved a complete response six cycles of treatment.

However, 3 years later, the patient presented with a new relapse (abdominal lymph node enlargement, new mass in the left orbit), but he refused any treatment. One month later, he complained of weakness in double limbs with normal muscle strength. Cranial MRI showed enlarged mass in the left orbit and abdominal lymph nodes were found in MRI (Figure 5D,E). The CNS involvement by Hodgkin lymphoma was confirmed with CSF cytology, and RS cells were found (Figure 4C).

He was treated with combination of chemotherapy for two cycles of MTX+PD1, finally achieving a partial response.

## 4 DISCUSSION

Because of the rarity in CNS involvement, the knowledge of these patients' clinical characteristics is derived from few case reports and small case series. As far we know of, there are only two reported case series of intracranial involvement of HL. From these findings, we can partially conclude that CNS involvement in HL is slightly more frequent in male patients during the fifth decade of life and it usually is of nodular sclerosing subtype with clinical stage III–IV. In our study, the four patients had clinical stage III–IV (Table 1).

**TABLE 1:** Characteristics of the patients described.

Article type	Sex	Median age/age (year)	Type of HL	Initial staging	B symptoms	Isolated CNS involvement or concurrent systemics	Neurological symptoms	CNS location	Author
Brief report (16 cases)	7 F, 9 M	45	CHL NOS*7 NS*7 MC*2	I–II*6 III–IV*9 Unknow*1	5	Isolated*5 Systemics*11	Pain/sensory*5 Weakness*3 Confusion*3 Headache*3 Seizure*3	Dural/meningeal*6 Frontal lobe*2 Parietal lobe*2 Temporal lobe*2 Cerebellum *1 Spinal cord*1	Elizabeth, et al.
Research (21 cases)	8F, 13 M	40	NS*16 MC*3 LP*1 Unknown*1	I–II*9 III–IV*11 Unknown*1	11	Isolated*4 Systemic*15 Unknown*2	Weakness*7 Headache*5 Sensory*4 Confusion*3 Aphasia/ Ataxia*1	Dural/meningeal*17 Cortical*12 Cerebellum*4 Spinal cord*4 Brainstem*3 Basal*2 Ganglia medulla*2	Chan, et al.
Case report	F	29	MC	II	1	Systemic	Headache and Blurred vision	Meningeal	Heidi Mociková, et al.
Case report	F	42	MC	IV	1	Isolated	Dysarthria, Head	Parietal lobe	Dioselina et al.
Case report	F	79	CHL NOS	IV	1	Unknown	Dysphagia, Fever	Meningeal	Sara, et al.
Case report	M	12	Unknown	IV	1	Systemic	Paralysis of eye, Nystagmus	Right midbrain	Vinayak, et al.
Case report	M	38	Unknown	I	1	Unknown	Ataxia	Fourth ventricular	Ahmad, et al.
Cases report (3 cases)	2F, 1 M	36	NS*1 LP*1 LD*1	I–II*1 III–IV*2	unknown	Isolated*2 Systemic*1	Headache*1 Dysphagia, Fever*1 Ataxia*1	Meningeal*2 Cerebellum*1	L. Gala, et al.
Cases report (4 cases)	3 M 1F	48.5	MC*2 LP*1 CHL NOS*1	III–IV*4	2	Isolated*3 Systemic*1	Dysarthria*2 Aphasia*1 Weakness*3	Left frontal*1 Brainstem*3 Cerebellum*1 Lateral ventricles*1 Infraorbital*1	Our study

Abbreviations: Age, age at diagnosis of CNS involvement; CHL‐NOS indicates classical Hodgkin's lymphoma not otherwise specified; CNS, central nervous system; F, female; LD, lymphocyte‐depleted classical Hodgkin lymphoma; LP, nodular lymphocyte predominant Hodgkin lymphoma; LR, lymphocyte‐rich classical Hodgkin lymphoma; M, male; MC, mixed cellularity classical Hodgkin lymphoma; NS, nodular sclerosis classical Hodgkin lymphoma.

The clinical presentation of CNS involvement by HL reported with mimicking of cerebral stroke symptoms. Common presentations include pain/sensory symptoms, weakness, cranial nerve palsies, altered mental status, headache, papilledema, coma, seizures and ataxia. The most common presenting feature was dural/meningeal‐based lesions in CNS involvement by HL.^3^, ^4^ Less often is the involvement of brain parenchyma, leptomeninges, cortical, cerebellum, spinal cord, and brainstem and pituitary^6^, ^7^ as shown in Table 1. Our patient showed involvement of the brainstem, cerebellum, and meninges at the time of relapse. The clinical presentation in our series included dysarthria, aphasia, weakness, and focal seizures. To our surprise, one case had tumour and ischemic stroke focus simultaneously. It may be related to trousseau's syndrome, which could promote cerebral infarction. Almost all patients with CNS involvement by HL had stroke symptoms, and it could be easily misdiagnosed as stroke. However, abnormal cerebrospinal fluid reminded us of the importance to distinguish stroke and CNS involvement. Moreover, intracranial involvement should be considered in any patient with known HL, who develops neurological complications, especially if there is dissemination of disease or relapse.

Diagnosis was most frequently established by either stereotactic or excisional biopsy. In Chan Y.et al study diagnostics were documented in 11 cases of which only five were considered positive and one case was diagnosed by CSF microscopy only, a spinal tap, nevertheless constitutes a feasible way of evaluating CNS involvement in symptomatic HL patients.^4^ In Elizabeth et al. study, CSF diagnostics was performed in nine patients and two patients had atypical cells in cerebrospinal fluid. A robust conclusion of potential strengths and limitations of the different diagnostic procedures should not be drawn due to the small number of patients and lack of CSF assessment in nearly half of patients.^3^ Due to the high cost of surgery and biopsy, there were limitations when used in patients with intracranial lesion. Lumbar puncture was performed to diagnosis in patients with CNS involvement by a cheaper and lesser invasive way in our case series. We were unable to perform a central review of cerebrospinal fluid cytology with positive immunohistochemistry staining for CD30 in two cases, because of insufficient cerebrospinal fluid specimen. Hence, it is our limitation with regards to diagnosis in these cases. Nevertheless, all the CNS histology samples were investigated by experienced hematopathologists at our academic cancer centers and were determined to be HL. Therefore, any patients presented with at least one neurological symptom or unexplained neurological signs should be evaluated carefully in a patient with diagnosis of HL.^4^ There is conflicting information in the literature regarding other possible risk factors, including suggestions of increased risk with a family history of HL, immune compromised, and EBV infection.^6^, ^7^ Pathophysiological mechanisms of CNS in HL remain incompletely understood. The proposed hypotheses include hematogenous spread through the bony skull or dura; or by direct extension meningeal involvement and metastasis.^8^, ^9^ Another theory is hematogenous dissemination. It is suggested that the large size of the Reed Sternberg (RS) cells may preclude their passage to the perivascular space of the CNS, and further filtering through the lung cells led to the rarity of intracranial involvement of HL.^10^ However, RS cells may secrete cytokines to weaken the blood–brain barrier, together with the other, tumour internal environment factors, contributing to CNS involvement.

Given the involvement of the skull and meninges in our patient suggested by MRI scan, as well as the location of her brain lesion, the mechanism of spread to the CNS, in this case, was likely contiguous hematogenous spread in Case 1 and Case 3, and meningeal involvement in the case two.

CNS disease in HL have been described in patients on initial presentation, after the diagnosis of disseminated disease, and, most commonly, with relapsing disease.^3^, ^5^, ^10^, ^11^ However, CNS HL has also been seen in patients after achieving complete remission after therapy.^9^ In a recent review of 21 CNS involvement patients, CNS HL had a feature of relapsed/refractory disease in 11 patients (52%), the median time from initial diagnosis of HL to development of CNS involvement was 1.9 years. 10/21 (48%) of patients had CNS HL discovered simultaneously with or prior to systemic disease, with an estimated crude incidence rate of 0.03% in all cases of HL.^4^

The infrequency of this disease makes information on therapeutic decision‐making difficult. There is a wide variety of treatment modalities that can be used, including guided radiotherapy alone, whole‐brain radiation, multiagent systemic chemotherapy and total or subtotal surgical resection.^3^, ^4^, ^12^, ^13^ The satisfactory response can be obtained in most patients after radiotherapy combined with systemic chemotherapy and brain resection (Table 2). Additional modalities described include intrathecal chemotherapy, and stem cell transplantation, especially in the face of meningeal involvement.^7^, ^11^ However, CNS involvement as a feature of relapsed/refractory disease was adversely prognostic, with the median PFS and OS were 7.6 and 29 months, respectively.^4^ Recently, in a phase 3 trial, a combination of brentuximab vedotin (an anti‐CD30 antibody‐drug conjugate) plus standard chemotherapy showed an improvement in the risk of progression, death, or incomplete response in patients with advanced stages of HL during a 2‐year follow‐up.^14^ However, there is still insufficient data to recommend the routine use of any specific treatment modality to prevent CNS involvement at the time of initial HL treatment, and more data are required to identify specific populations in whom this could be beneficial.

**TABLE 2:** Treatment of the patients from case series by Elizabeth R, et al and Chan Y.et al study.^3^, ^4^

Patients	Method of diagnosis	Radiotherapy	Chemotherapy	Assessment
1	Biopsy	None	HD‐MTX, cytarabine, thiotepa	CR
2	Biopsy	Cord, 30 Gy in 15	Prednisone, intratheca, MTX	CR
3	Biopsy	Cord, 30 Gy in 20	ABVD	CR
4	Biopsy	Largest lesions	BEACOPP	CR
5	Biopsy	WBRT, 30 Gy in 10	Unknown	CR
6	Resection	WBRT (35 Gy)	None	CR
7	Biopsy	PBRT (36 Gy)	Stamford V+PBSCT+MTX	CR
8	Resection	PBRT (36 Gy)	ABVD+thiotepa	CR
9	Resection	None	BVAM, BEAM+PBSCT+MTX	CR
10	Biopsy	WBRT (36 Gy)	Ifosfamide	CR
11	Biopsy	WBRT (45 Gy)	MOPP	CR
12	Biopsy	None	Rituximab, IVAC, high‐dose MTX	CR
13	Resection	WBRT (10.8 Gy with 23.4 Gy boost)	None	CR
14	Biopsy	None	Bonner Protocol	CR
15	CSF	WBRT+cord	Dexamethasone, cytarabine, cisplatin	PR
16	Biopsy	WBRT, 30 Gy in 10	Intrathecal liposomal, cytarabine	PR
17	Biopsy	WBRT	MVPP	PR
18	CSF	Radiosurgery	ABVD	PR
19	Biopsy	Radiosurgery	None	PR
20	Biopsy	WBRT, 40 Gy in 20	ESHAP	SD
21	Biopsy	None	None	PD
22	Biopsy	Cord, 30 Gy in 20	Rituximaba, ifosfamide, etoposide, cytarabine	PD
23	Biopsy	None	Dexamethasone+BV	PD
24	Autopsy	None	Lomustine, procarbazine, prednisone, cytoxan+cytarabine	PD
25	Biopsy	Unknown	Unknown	Uknown
26	Biopsy	Unknown	Unknown	Unknown
27	Biopsy	Unknown	Unknown	Unknown
28	Biopsy	Unknown	Dexamethasone，ICE	Unknown
29	Resection	None	BEACOPP	NA
30	Resection	None	None	NA
31	Resection	WBRT, 40 Gy in 20	None	NA
32	Resection	None	Dexamethasone	NA
33	Resection	WBRT, 40 Gy in 10	Bv+bendamustine+fosfamide+mitoxantrone	NA
34	Resection	WBRT, 40 Gy in 20	Dexamethasone	NA
35	Resection	WBRT, 20 Gy in 10	None	NA
36	Resection	WBRT (to begin)	None	NA
37	Resection	None	None	NA

Abbreviations: ABVD, doxorubicin, bleomycin, vinblastine, dacarbazine; BEACOPP, bleomycin, etoposide, doxorubicin, cyclophosphamide, vincristine, prednisone, procarbazine; BEAM, carmustine, etoposide, cytarabine, melphalan; PBSCT, peripheral blood stem cell transplantation; BV, brentuximab vedotin; BVAM, carmustine, vincristine, cytarabine, and methotrexate; CEP, lomustine, etoposide, prednisone; CR, complete response; DHAP, dexamethasone, cytarabine, cisplatin; EHSAP, etoposide, methylprednisone, cytarabine, cisplatin; HD, high‐dose; ICE, ifosfamide, carboplatin, etoposide; IVAC, ifosfamide, etoposide, and high‐dose cytarabine; MOPP, mechlorethamine, vincristine, procarbazine, prednisone; MTX, methotrexate; MVPP, mustine, vinblastine, procarbazine, prednisone; NA, not assessed; PBRT, partial brain radiation therapy; PD, progressive disease; PR, partial response; SD, stable disease; WBRT, whole brain radiation therapy.

In our study, all patients underwent second‐line chemotherapy without radiotherapy. The third and forth case were treat with PD1 resulting in partial responses (PR). Recent research has found overexpression of programmed death‐1 (PD‐1) ligands, including PD‐L1 on RS cells. PD‐L1/PD‐L2 alterations are a defining feature of HL and result in very high expression of PD‐L1 or PD‐L2 on the cell surface, thereby protecting RS cells from T‐cell mediated killing. Hence, PD1 is now being incorporated into the frontline therapy.^15^ Various case series studies by Elizabeth, et al and Chan.et al, showed that more patients achieved complete response (CR) after chemotherapy and radiotherapy.^3^, ^4^ Unfortunately, treatment with PD1 and radiotherapy has not been reported in the patients with CNS involvement due to the rarity of CNS involvement in HL.

## 5 CONCLUSION

CNS involvement in HL is rare, and we report four patients with relapsed HL with CNS involvement. Tissue diagnosis and cerebrospinal fluid cytology analysis is essential for any patient with known HL presenting with a focal CNS lesion or a new unexplained neurological pathology. The clinical course, outcome, and treatment of patients with CNS involvement needs more attention in the clinical setting.

## AUTHOR CONTRIBUTIONS

**Xiping Liang:** Conceptualization (equal); data curation (equal); investigation (equal); methodology (equal); validation (equal); writing – original draft (equal). **Chaoyu Wang:** Conceptualization (equal); data curation (equal); formal analysis (equal); methodology (equal); writing – original draft (equal). **Yao Liu:** Conceptualization (equal); data curation (equal); supervision (equal); validation (equal); writing – review and editing (equal).

## FUNDING INFORMATION

The study was supported in part by the National Natural Science Foundation of China (Grant no. 81670100).

## CONFLICT OF INTEREST STATEMENT

The authors declare that they have no competing interests.

## Data Availability

The raw data supporting the conclusions of this article will be made available by the authors, without undue reservation.
